# Comparative Expression Profiling of Snf2 Family Genes During Reproductive Development and Stress Responses in Rice

**DOI:** 10.3389/fpls.2022.910663

**Published:** 2022-05-31

**Authors:** Mingliang Guo, Heming Zhao, Zhimei He, Wenchao Zhang, Zeyuan She, Mohammad Aqa Mohammadi, Chao Shi, Maokai Yan, Dagang Tian, Yuan Qin

**Affiliations:** ^1^State Key Laboratory of Ecological Pest Control for Fujian and Taiwan Crops, College of Plant Protection, Fujian Agriculture and Forestry University, Fuzhou, China; ^2^Center for Crop Biotechnology, College of Agriculture, Anhui Science and Technology University, Fengyang, China; ^3^College of Agriculture, Fujian Agriculture and Forestry University, Fuzhou, China; ^4^State Key Laboratory for Conservation and Utilization of Subtropical Agro-Bioresources, Guangxi Key Lab of Sugarcane Biology, College of Agriculture, Guangxi University, Nanning, China; ^5^College of Horticulture, Fujian Agriculture and Forestry University, Fuzhou, China; ^6^Fujian Provincial Key Laboratory of Haixia Applied Plant Systems Biology, College of Life Sciences, Fujian Agriculture and Forestry University, Fuzhou, China; ^7^Fujian Provincial Key Laboratory of Genetic Engineering for Agriculture, Biotechnology Research Institute, Fujian Academy of Agricultural Sciences, Fuzhou, China; ^8^Pingtan Science and Technology Research Institute, Fujian Agriculture and Forestry University, Fuzhou, China

**Keywords:** rice (*Oryza sativa* L.), Snf2 family, biotic and abiotic stress, *OsCHR726*, salinity stress

## Abstract

Sucrose non-fermenting 2 (Snf2) protein family, as chromatin remodeling factors, is an enormous and the most diverse protein family, which contributes to biological processes of replication, transcription, and DNA repair using the energy of adenosine triphosphate (ATP) hydrolysis. The members of Snf2 family proteins have been well characterized in Arabidopsis, rice, and tomato. Although this family received significant attention, few genes were identified uniquely for their roles in mediating reproductive development and stress tolerance in rice. In the present study, we comprehensively analyzed the expression profiling of Snf2 genes during reproductive development and biotic/abiotic stresses. Our results showed that five proteins (OsCHR712/715/720/726/739) were mainly localized in the nucleus, while OsCHR715/739 were also slightly expressed in the cell membrane. There were abundant *cis*-acting elements in the putative promoter of Snf2 genes, including dehydration, MeJA, MYB binding site for drought, ABA-responsive, and stress-responsive element. Most of the genes were induced immediately after *Magnaporthe oryzae* infection at 12 h post-infection (hpi). About 55% of the total genes were upregulated under salt and drought stresses during the entire time, and 22–35% of the total genes were upregulated at 3 h. It was noteworthy that the seven genes (*OsCHR705*, *OsCHR706*, *OsCHR710*, *OsCHR714*, *OsCHR721*, *OsCHR726*, and *OsCHR737*) were upregulated, and one gene (*OsCHR712*) was downregulated under salt and drought stresses, respectively. The deficiency of *OsCHR726* mutations displayed a hypersensitive phenotype under salt stress. These results will be significantly useful features for the validation of the rice Snf2 genes and facilitate understanding of the genetic engineering of crops with improved biotic and abiotic stresses.

## Introduction

Sucrose non-fermenting 2 (Snf2) family proteins consist of a sizeable and diverse class of multiprotein assemblies that remodel chromatin complexes to contribute to the biological processes of replication, transcription, and DNA repair using the energy of ATP hydrolysis. Chromatin remodeling factors (CHRs) are ATPases from the Snf2 family and modulate the position of nucleosomes on chromatin to enable dynamic access to packaged DNA (Song et al., 2021). During plant stress responses, altered transcriptional responses have been linked to chromatin-mediated inducible gene expression, which is performed by covalently modifying histone and/or the DNA, or non-covalently altering the nucleosome position, conformation, occupancy, and composition by chromatin remodeling ATPases (Li et al., 2007; Chinnusamy and Zhu, 2009; Kim et al., 2010). Snf2 family proteins, especially in a plant, are often classified by two conserved domains (SNF2_N/DExx and Helicase_C/HELICc) in the helicase-like region (Flaus et al., 2006; Knizewski et al., 2008). The Snf2 family members are identified and characterized as 41, 40, and 45 proteins in model plants *Arabidopsis*, rice, and tomato, respectively, and classified into 24 subfamilies of six groups (Song et al., 2021). The corresponding chromosome location, phylogenetic relationship, domain architectures, and expression pattern of partial genes have been clearly described (Knizewski et al., 2008; Hu et al., 2013; Zhang et al., 2019).

Many publications have insight into the critical roles of CHRs in regulating development, growth, and stress response in general plants (Han et al., 2015; Sarnowska et al., 2016; Song et al., 2021). As sessile organisms, the plants must depend on their capacity to cope with the adverse environment or situation, such as biotic stresses (pathogenic infections) and abiotic stresses (drought, salt stress, extreme temperatures, and heavy metals elements; Mohammadi et al., 2020; Han et al., 2021). When first exposed to stress, plants initiate rapid gene expression modulation by altering chromatin structures at promoters and other regulatory DNA regions using chromatin-remodeling enzymes (Mlynarova et al., 2007). *MINU1/CHR12*, an Snf2/Brahma-type chromatin-remodeling gene, participates in mediating the temporary growth arrest in *Arabidopsis*. The overexpression of *MINU1/CHR12* leads to growth retardation under adverse stress conditions, and *MINU1/CHR12* loss-function-of mutant displays tolerance to salt, drought, and heat stresses (Mlynarova et al., 2007). *BRM* (*CHR2*, an Snf2 subfamily member) directly represses the transcription of *ABI5*, and loss-of-function of *BRM* causes ABA hypersensitivity during post-germination development. In addition, *brm* was reported to increase drought tolerance (Han et al., 2012). *PICKLE* (*PKL/CHR6*, a Mi-2/CHD3 subfamily member) activates the expression of auxin and cell elongation-related genes *IAA19* (*INDOLE-3-ACETIC ACID INDUCIBLE 19*) and *IAA29* by repressing H3K27me3 deposition. The mutant of *PKL* displays hypocotyl reduction (Zha et al., 2017). In addition, a recent report shows that *pkl* mutant is hypersensitive to cold and freezing stresses (Yang et al., 2019).

Besides *Arabidopsis*, several Snf2 family proteins have been studied during biotic or abiotic stresses in other plant species. For example, the loss-function-of mutant of *ZmCHB101* (the ortholog gene of SWI3D in *Arabidopsis*) is dramatically sensitive to osmotic and salt stress, and the transcriptional level of stress-responsive genes are affected by regulating RNA polymerase II association and nucleosome density near TSS (transcription start site) in the maize (Yu et al., 2018). Under salt stress, *SlCHR1*, the ortholog gene of *MINU* in Snf2 family, regulates the growth retardation in tomato, similar to *CHR12* in *Arabidopsis*. *OsCHR4/CHR729*, a CHD3/Mi-2 subfamily member, affects the development of seedling and root via the signaling pathways of gibberellin and auxin, respectively, and decreases the contents of chloroplast in adaxial mesophyll cells in rice (Hu et al., 2012; Ma et al., 2015a; Wang et al., 2016). Functional deficiency of *OsRFS* (*Rolled Fine Striped*) affects leaf rolling, width, chloroplast development, reactive oxygen species (ROS) scavenging, and *osrfs* mutants exhibited accumulation of ROS due to the loss of H3K4me3 at the genomic loci of ROS-related genes (Cho et al., 2018; Mohammadi et al., 2021). *OsALT1* (*Alkaline Tolerance 1*, *OsCHR706*), a Ris1 subfamily chromatin-remodeling ATPase, improves tolerance to alkaline stress by reducing ROS levels and alleviating oxidative stress damage (Guo et al., 2014).

Snf2 family proteins, as CHRs, is an enormous and most diverse protein family in rice. Previous researches have shown the function of some genes, such as *OsCHR4*, *OsRFS*, *OsALT1, OsDDM1*, *OsCHR721*, *OsBRHIS1*, *OsENL1* (Higo et al., 2012; Hu et al., 2012; Guo et al., 2014; Hara et al., 2015; Li et al., 2015; Ma et al., 2015a; Wang et al., 2016; Cho et al., 2018; Zhang et al., 2020b). The systematical expression profiles of Snf2 family genes have not been analyzed during reproductive development and biotic/abiotic stresses. This study deeply investigated the expression profiling of the rice Snf2 genes in different organs/tissues, as well as the prediction of *cis*-acting elements in the promoter regions and responses under drought and salt treatments. Moreover, we report that the mutant lines of *OsCHR726*, which encodes a SMARCAL1 CHR, are sensitive to salinity stress. The results extend our understanding of Snf2 genes in plant development and are a valuable resource for further investigating stress tolerance in rice.

## Materials and Methods

### Plant Materials and Growth Conditions

The *OsCHR726* T-DNA insertion mutant (PFG_2C-10296) in the Dongjin (*Oryza sativa* L.) background was obtained from Kyung Hee University, South Korea. The detailed information on the mutant can be searched at the SIGnAL database^1^. The *OsCHR726* Clustered Regularly Interspaced Short Palindromic Repeats/CRISPR-associated proteins 9 (CRISPR/Cas9)-mediated mutant lines used in this study were in Zhonghua 11 (ZH11, *Oryza sativa* L.) background, which also was used as wild-type for analysis of Snf2 genes expression in specific tissues, as well as under biotic and abiotic stresses. All rice plants were grown in the greenhouse at Fujian Agriculture and Forestry University at 22–32°C and 80–90% humidity with a 14 h/10 h (light/dark) photoperiod.

To analyze the expression profile of representative Snf2 genes during vegetative and reproductive development, the tissues for expression pattern analysis were: 7-day-old seedling (YS), root (YR), and leaf (YL); 70-day-old leaf (ML); 0–3 cm panicles (P1); 3–5 cm panicles (P2); stigma (Sti) of mature ovary (OV) before pollination; and seed at 25 DAP (S5, day after pollination). The stamens and pistils at different developmental stages were carefully collected from young panicles of 60–75-day-old seedlings. Stamens and pistils from the different spikelets with 2–3, 3–4, 4–5, 5–6, 6–7 mm length and before flowering were defined as An1–6 and Ov1∼6, respectively.

For expression analysis of Snf2 genes in response to ABA, IAA, and other stress treatments, the seeds were germinated and sown on a plastic net floating on a nutrient solution in a growth chamber (Yoshida et al., 1971; Zhao et al., 2015). The seedling roots of 14-day-old ZH11 were submerged into a nutrient solution containing 200 mM NaCl, 100 mM ABA, 150 μM IAA, and nutrient solution treatment, respectively. For cold testing, the seedlings were treated at 4°C and the seedlings were carefully transferred onto paper as drought stress, in which they were air-dried (Zhao et al., 2012). The samples were collected at 0, 1, 3, 6, 12, and 24 h of H_2_O, salt, ABA, cold, and drought stresses. For biotic stress, 2-week-old seedlings were sprayed with a spore suspension (1 × 10^5^ spores/mL) of the *Magnaporthe oryzae* (*M. oryzae*) isolate Guy11, and the leaf samples were taken 12, 24, 36, 48, 60, and 72 h post-infection (hpi), respectively. All materials contained three biological replicates and were immediately frozen in liquid nitrogen and stored at –80°C for RNA extraction.

### Expression Data Analysis of Snf2 Family in Rice

The microarrays data were extracted from the Rice Functional Genomic Express Database (see footnote 1) and used to analyze the expression profiles of Snf2 genes in various tissues during different development stages (GSE6893, GSE6901, GSE7951; Ma et al., 2011). The microarray data of Sti and OV were from GSE7951 and YS was from GSE6901. The GSE6893 included the data of YR, ML, YL, SAM, P1-P6, and S1–S5. The data is provided in Supplementary Table 1. For microarray analysis, if more than one probe set was available for one gene, the average of the values was used for further study. The relative expression levels of Snf2 genes were determined by extracting their respective data from the total expression matrix using Genesis software.

The heatmaps were presented on expression data of Snf2 genes to drought and salt using the R package pheatmap. A lot of values are concentrated in a very small range, and suddenly there are a few very large values that are disproportionately large. The result is that most of the differences between the values are obscured to accommodate the few extreme values, leaving large areas of almost the same color. Standardization is to retain the law of data and scale the value in a relatively stable range after a certain proportion control, which is more convenient for showing.

### RNA Isolation and qRT-PCR Analysis

Total RNA of all collected samples was extracted using Plant RNeasy Mini kit (Qiagen, Hilden, Germany) according to the manufacturer’s instructions. A total of 1 μg of RNA was reverse transcribed using the PrimeScript RT-PCR kit (Takara, Kyoto, Japan; Cai et al., 2019). The relative expression level was detected by quantitative real time PCR (qRT-PCR) using the Bio-Rad QRT-PCR system (Foster City, CA, United States) and SYBR Premix Ex TaqII (TaKaRa Perfect Real Time; Zhang et al., 2020a). The qRT-PCR program was: 95°C for 30 s; 40 cycles of annealing at 95°C for 5 s, and extension at 60°C for 35 s; 95°C for 15 s (Zhang et al., 2020a; Zhao et al., 2021). The rice *OsUBQ5* gene was used as an internal control (Jain et al., 2006). To evaluate the relative expression levels of the examined genes, we used the comparative ΔΔC_*T*_ method (Su et al., 2017). The gene-specific primers are listed in Supplementary Table 2.

### Subcellular Localization Analysis of OsCHRs

The full-length coding sequences of *OsCHR712/715/720/726/739* were amplified from WT cDNA using the primers listed in Supplementary Table 3. The PCR fragments were cloned into the pENTER/D-TOPO vector (Invitrogen, CA, United States), and pENTER/D-TOPO clones were recombined into the pGWB506 vector using LR ClonaseII enzyme (Invitrogen). The 35S:CDS-GFP recombinant construction and 35S:GFP (vector control) were transformed into *Agrobacterium tumefaciens* (GV3101) and infiltrated into tobacco leaves. The fluorescence signals were observed using a confocal microscope (SP8, Leica, Germany), and the excitation wavelength was 488 and 405 nm.

### Analysis of the Putative Promoter Regions of Snf2 Genes

The *cis*-acting promoter elements are crucial for the coordinated expression of genes. We retrieved ≤ 2 kb upstream sequence of all 40 Snf2 genes and predicted the critical regulatory elements responsible for biotic and abiotic stresses by PlantCARE (Lescot et al., 2002).

### Identification of the T-DNA Insertion and CRISPR/Cas9-Mediated Mutations

The T-DNA insertion in *oschr726* was confirmed by PCR using primers 2C-10296-Lp and 2C-10296-Rp, and the T-DNA-specific primer LB (2717). In addition, we generated a gRNA construct with the gRNA (5′GCCGAGGGCTTCGCCTACCCCGG 3′), and plant-optimized Cas9 driven by rice *OsU6a* and maize *pUBI-H* promoters, respectively (Ma et al., 2015b). The plasmid was transformed into WT ZH11 callus, and the DNA isolated from transgenic plant leaves was amplified by PCR and sequencing analysis using the primer set P1 and P2. The primers sequences are described in Supplementary Table 8 for genotyping identification. The gene structure of *OsCHR726* was searched from Gene Structure Display Server^2^.

### Salt Stress of *oschr726* Mutant Lines

To investigate the mutant salt response in a solid medium, seeds were disinfected with hypochlorous acid and germinated on half MS (Murashige and Skoog) medium for 2 days then transplanted to half MS with 0, 100, 150 mM NaCl for 5 days at 28°C in the growth chamber with a 14 h/10 h (light/dark) photoperiod.

## Results

### Expression Analysis of Snf2 Genes in Various Tissues Using Microarray Data

Snf2 family proteins, as CHRs, consisting of enormous protein members, play a crucial role in the process of plant development and growth, including transcription, replication, homologous recombination, and DNA repair. (Han et al., 2015; Sarnowska et al., 2016; Ojolo et al., 2018; Singh et al., 2020). The protein members of the Snf2 family have been well-characterized in rice (Hu et al., 2013). However, the spatial expression profiles of Snf2 family genes have not been analyzed.

**FIGURE 1:**
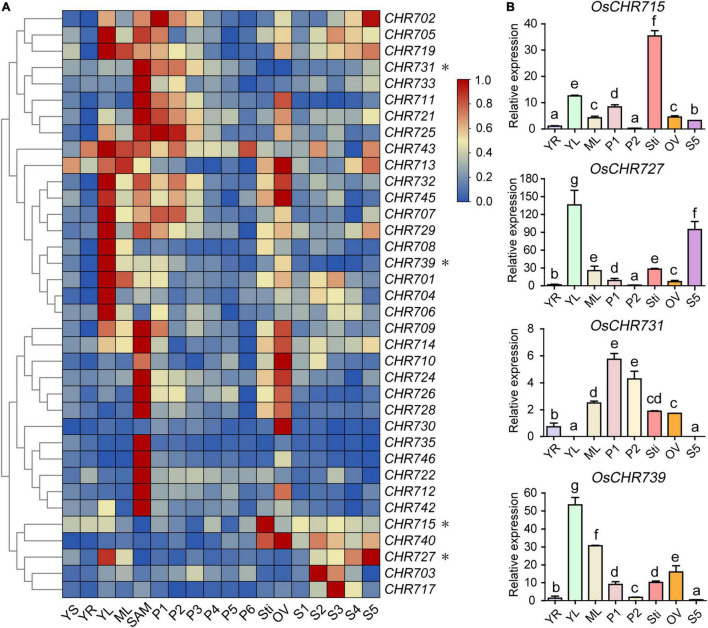
Expression profiles of Snf2 genes in various organs. **(A)** The microarray data (GSE6893, GSE6901, and GSE7951) of Snf2 genes’ expression in various organs at different developmental stages were reanalyzed. A heat map representing hierarchical clustering of average log2 expression values of Snf2 genes in various organs are generated (YS, 7-day-old seedlings; YR, roots from 7-day-old seedlings; YL, leaves from 7-day-old seedlings; ML, mature leaf; SAM, shoot apical meristem; different stages of panicle development: P1, 0–3 cm; P2, 3–5 cm; P3, 5–10 cm; P4, 10–15 cm; P5, 15–22 cm; P6, 22–30 cm; Sti, stigma of mature ovaries; OV, mature ovary; different stages of seed development: S1, 0–2 dap (day after pollination); S2, 3–4 dap; S3, 5–10 dap; S4, 11–20 dap; S5, 21–29 dap). The representative Snf2 genes are differentially expressed in various organs, for which real-time PCR analysis was performed, indicated by an asterisk (*) on the right. The color scale (representing average log signal values) is shown on the right side. **(B)** The representative Snf2 genes (asterisks indicate genes in **A**) are differentially expressed in various organs consistent with the samples of microarray using the qRT-PCR. Error bars indicate standard deviations of independent biological replicates (*n* = 3). Different letters denote significant differences at *P* < 0.05 according to ANOVA in combination with Duncan’s multiple range test.

To this end, the microarray data of various tissues were collected during vegetative and reproductive developmental stages from the Rice Functional Genomic Express Database (see footnote 1), including 7-day-old seedlings (YS), young root (YR), leaf (YL), mature leaf (ML), shoot apical meristem (SAM), panicles (P1-P6), stigma (Sti) of mature ovary (OV), and seed (S1–S5) development. The result showed that a total of 36 (90%) Snf2 genes were expressed in different tissues, while the rest of four genes (*OsCHR720*, *OsCHR736*, *OsCHR737*, *OsCHR741*) were not detected in the microarray data (Supplementary Table 1). A hierarchical cluster display average of log signal values of these genes was produced (Figure 1A). The microarray analysis revealed that 15 (37.5%) and 20 (50%) of Snf2 genes were predominately expressed in YL and SAM, respectively. *OsCHR735*, *OsCHR746*, *OsCHR722*, and *OsCHR742* were distinctively expressed in SAM, and *OsCHR704*, *OsCHR706* were uniquely expressed in YL. The expression levels of 13 (32.5%) Snf2 genes were highly expressed in OV. *OsCHR731* and *OsCHR733* are highly expressed in SAM, P1, and P2, while *OsCHR711*, *OsCHR721*, *and OsCHR725* were highly expressed in SAM, P1, P2, and OV. Interestingly, *OsCHR730*, *OsCHR715*, *OsCHR703*, and *OsCHR717* were uniquely expressed in OV, Sti, S2, and S3, respectively. It was noteworthy that the four genes (*OsCHR715*, *OsCHR727*, *OsCHR731*, and *OsCHR739*) marked with asterisks in Figure 1A were confirmed to be specifically expressed in Sti, YL and S5, P1 and P2, and YL using the qRT-PCR, respectively, which agreed well with the microarray data (Figure 1B). Taken together, we can conclude that most of the Snf2 genes were predominately expressed in YL, SAM, and OV, suggesting the participation of Snf2 genes in the development of young tissues and reproductive organs.

### Most Snf2 Genes Are Highly Expressed in the Late Development Stages of Stamen and Pistil

**FIGURE 2:**
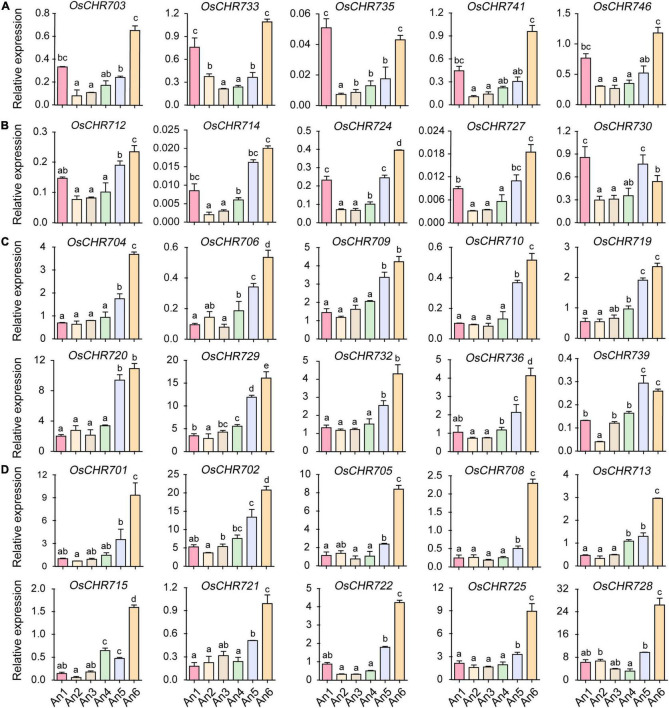
The relative expression level of Snf2 gene family in the stamen. **(A)** Predominately expressed Snf2 genes at the An1 and An6 stages. **(B)** Preferentially expressed Snf2 genes at the An1, An5, and An6 stages. **(C)** Preferentially expressed Snf2 genes at the An5 and An6 stages. **(D)** Predominately expressed Snf2 genes at the An6 stages. An1-5, the stamen of 2–3, 3–4, 4–5, 5–6, and 6–7 mm spikelet, respectively; An6, the stamen in the spikelet before flowering. The *y*-axis is the relative expression level of the gene compared to *OsUBQ5* in different developmental stages of stamen using the qRT-PCR. The relative expression level of *OsCHR701* to *OsUBQ5* in An1 stage normalized as “1” was used as reference. Error bars indicate standard deviations of independent biological replicates (*n* = 3). Different letters denote significant difference at *P* < 0.05 according to ANOVA in combination with Duncan’s multiple range test.

It has been reported that *OsCHR721*, a nuclear protein belonging to the SMARCAL1 subfamily in the Snf2 family, played a crucial role in both male and female reproductive development (Zhang et al., 2020b). To investigate the temporal expression profile of Snf2 family genes and find the candidate key genes that may have functions during the development of reproductive organs in rice, we performed a more comprehensive analysis of stamen and pistil at specific developmental stages using qRT-PCR. According to the length of spikelets, the stamens and pistils were divided into six developmental stages (An1–6 and Ov1–6). *OsCHR703*, *OsCHR733*, *OsCHR735*, *OsCHR741*, and *OsCHR746* showed similar expression patterns, that is, they were preferentially expressed in both the early (An1) and late (An6) developmental stages of stamen (An1, spikelet length = 2∼3 mm, An6, mature spikelet, Figure 2A). *OsCHR712*, *OsCHR714*, *OsCHR724*, *OsCHR727*, *OsCHR730*, and *OsCHR743* were preferentially expressed in the An1, An5, and An6 developmental stages of stamen (Figure 2B and Supplementary Figure 1A). *OsCHR717* and *OsCHR737* were preferentially expressed in the An4–6 (Supplementary Figure 1B). *OsCHR704*, *OsCHR706*, *OsCHR709*, *OsCHR710*, *OsCHR719*, *OsCHR720*, *OsCHR729*, *OsCHR732, OsCHR736*, and *OsCHR739* were predominately expressed in the late developmental stages of stamen (An5 and An6, Figure 2C). There are several genes uniquely expressed in the An6, such as *OsCHR701*, *OsCHR702*, *OsCHR705*, *OsCHR708*, *OsCHR713*, *OsCHR715*, *OsCHR721*, *OsCHR722*, *OsCHR725*, and *OsCHR728* (Figure 2D). These results exhibited that most Snf2 genes were predominately expressed in the late development stages of stamen, and suggested that Snf2 genes might play an important role in mature stamen development.

**FIGURE 3:**
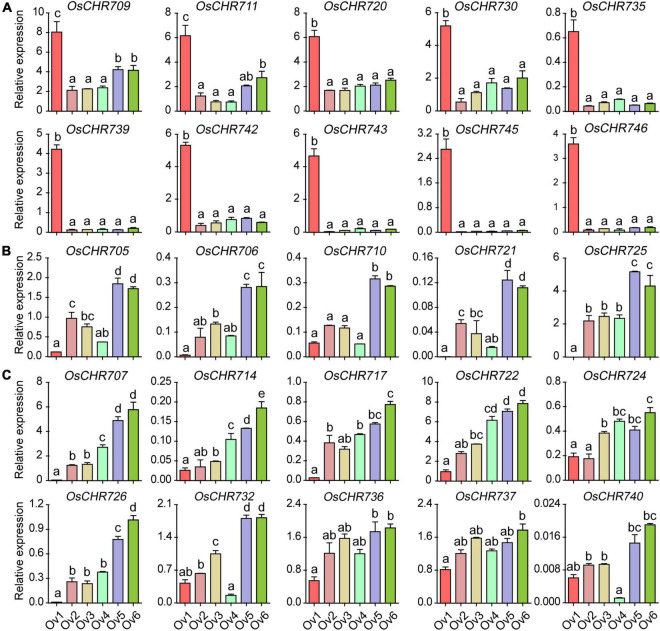
The relative expression level of Snf2 gene family in the ovary. **(A)** Specifically expressed Snf2 genes at the early developmental stages (Ov1) in the ovary. **(B)** Predominantly expressed Snf2 genes at the late developmental stages (Ov5 and Ov6) in the ovary. **(C)** The expression level of Snf2 gene gradually escalated from early to late developmental stages in the ovary. Ov1-5, the ovary of 2–3, 3–4, 4–5, 5–6, and 6–7 mm spikelet, respectively; Ov6, the ovary in the spikelet before pollination. The *y*-axis is the relative expression level of the gene compared to *OsUBQ5* in different developmental stages of the ovary using the qRT-PCR. The relative expression level of *OsCHR701* to *OsUBQ5* in An1 stage normalized as “1” was used as reference. Error bars indicate standard deviations of independent biological replicates (*n* = 3). Different letters denote significant differences at *P* < 0.05 according to ANOVA in combination with Duncan’s multiple range test.

Similarly, many genes displayed specific expression patterns during the pistil’s development. About 30 and 40% Snf2 genes were predominantly expressed in the early (Ov1, spikelet length = 2∼3 mm) and late (Ov6, mature spikelet) developmental stages of pistils, respectively. *OsCHR709*, *OsCHR711*, *OsCHR720*, *OsCHR730*, *OsCHR735*, *OsCHR739*, *OsCHR742*, *OsCHR743*, *OsCHR745*, and *OsCHR746* were specifically expressed in the Ov1 (Figure 3A). *OsCHR705*, *OsCHR706*, *OsCHR710*, *OsCHR721*, and *OsCHR725* were highly expressed in the late developmental stages of pistils (Ov5, spikelet length = 6–7 mm, Ov6, mature spikelet, Figure 3B), whereas, *OsCHR704*, *OsCHR712*, *OsCHR713*, *OsCHR715*, and *OsCHR727* were preferentially expressed in both early and late developmental stages of the pistils (Supplementary Figure 2A). *OsCHR703*, *OsCHR708*, *OsCHR719*, *OsCHR733*, and *OsCHR741* were predominately expressed in the Ov4 stages (Supplementary Figure 2B). *OsCHR707*, *OsCHR714*, *OsCHR717*, *OsCHR722*, *OsCHR724*, *OsCHR726*, *OsCHR732*, *OsCHR736*, *OsCHR737*, and *OsCHR740* showed similar expression patterns, and their expression level gradually increased with the developmental period (Figure 3C). However, other gene transcripts were abundant in stamen and pistils (Supplementary Figures 1C, 2C). These data indicated that these genes might play various roles in specific reproductive tissues at different development stages and provided better reference to explore the key regulatory genes for reproductive organ development in crops.

### OsCHRs Are Mainly Located in the Nucleus

**FIGURE 4:**
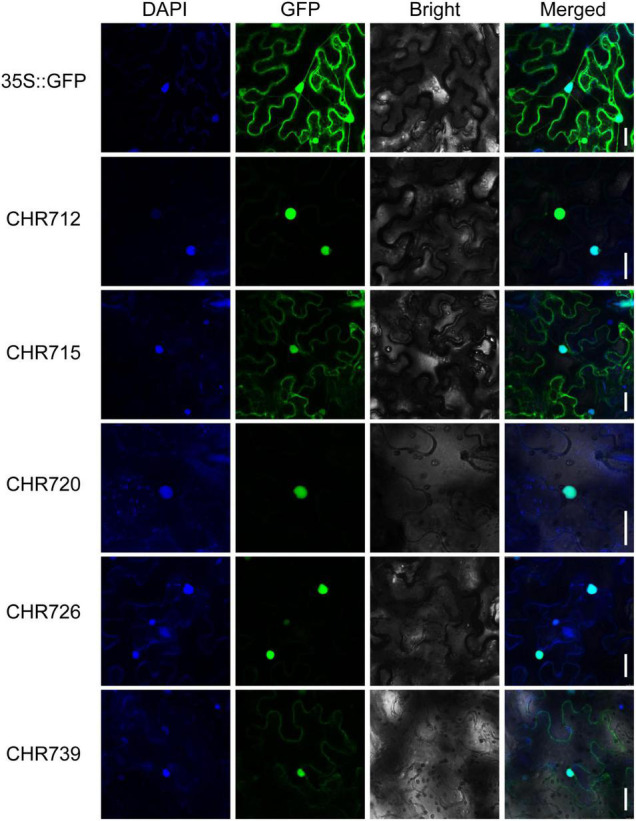
The subcellular localization of OsCHR712/715/720/726/739. The 35S:OsCHR712/715/720/726/739-GFP and control vector (35S:GFP) were transiently expressed in tobacco epidermal leaf cells. Confocal microscopy images were visualized after 24-h transformation. Bar = 30 μm.

Snf2 proteins, as CHRs, control many aspects of DNA events (Hu et al., 2013) and serve as an integrative platform that translates various signals from the cellular environment into regulated responses from DNA (Knizewski et al., 2008). To evaluate the subcellular localization of *OsCHRs* proteins, the full-length coding sequences of *OsCHR712/715/720/726/739*, which were from different subfamilies, were severally fused with the sequence encoding green fluorescence protein (GFP), and the vectors were expressed transiently in tobacco epidermal cells. As the results are shown in Figure 4, the fluorescence signal from 35:GFP vector was detected throughout the whole cell and co-localized with DAPI signal in the nucleus. Then the 35S:OsCHR712/715/720/726/739-GFP were co-localized with the DAPI signal, suggesting that these five proteins were mainly localized in the nucleus. Meanwhile, OsCHR715/739 were also slightly expressed in the cell membrane (Figure 4). Taken together, our results suggested that Snf2 genes might play crucial roles in the nucleus and correspond to the predicted function of the family proteins.

### Most Dehydration- and Drought-Responsive Elements Distributed in the Putative Promoter of Snf2 Genes

The *cis*-acting promoter elements are crucial for the coordinated expression of the genes. Then it is critical to characterize the regulatory elements to associate the expression profiles with the genetic components. We retrieved the promoter region (2 kb upstream sequence of the start codon) of all 40 Snf2 genes from the rice database. We predicted the *cis*-acting elements responsible for biotic and abiotic stress responses by PlantCARE (Lescot et al., 2002). A total of 11 stress-responsive elements were observed in the putative promoter regions of the Snf2 genes (Supplementary Table 4). These included DRE or binding site for MYC transcription factor for dehydration responsive elements (Narusaka et al., 2003; Tran et al., 2004). LTR for low temperature-responsive element, STRE or TC-rich motifs for stress responses, CCAAT box and MYB binding sites responsive to drought treatments, Box S and WUN motif for wounding and pathogen elicitation response elements (Xu et al., 2011; Yin et al., 2017). Several phytohormone responsive elements were also observed in the promoter regions, such as TCA element for salicylic acid responses, ERE as ethylene responses, CGTCA- or TGACG-motif for MeJA responsive element, ABRE as ABA-responsive element, TGA-element or AuxRR core or AuxRE for auxin responses, GARE-motif, and TATC-box for gibberellin responses.

**FIGURE 5:**
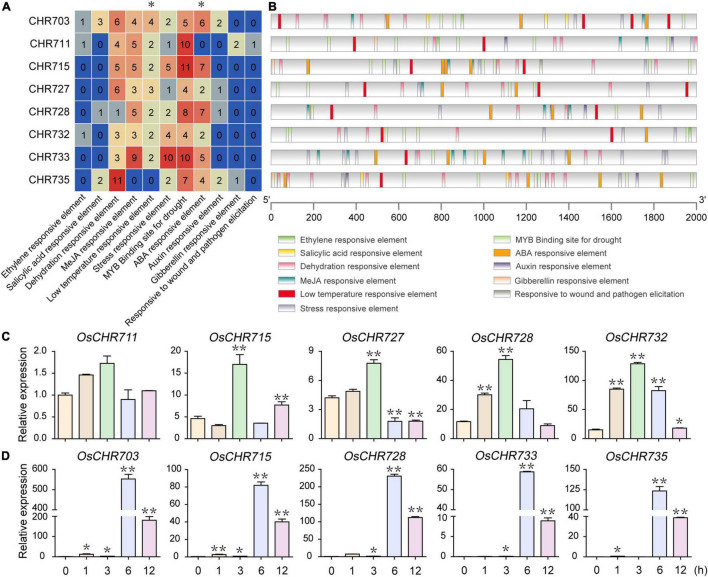
Prediction of *cis*-acting elements of environmental stress-related and hormone-responsive in Snf2 genes. **(A)** Number of *cis*-acting elements detected in the promoter regions (sequence retrieved from about ≤ 2 kb upstream region). The *cis*-acting elements were divided into 11 types. **(B)** Quantity, kind and position of environmental stress-related and hormone-responsive elements in the putative promoters of genes in Figure 5A. **(C)** The qRT-PCR analysis of Snf2 genes containing multiple LTR (low temperature-responsive) elements under 4°C treatment. **(D)** The qRT-PCR analysis of Snf2 genes containing multiple ABA-responsive elements under ABA treatment. The *y*-axis is the relative expression level of the gene compared to *OsUBQ5* under different treatments using the qRT-PCR **(C,D)**. The relative expression level of *OsCHR711* to *OsUBQ5* in an unstressed sample (0 h) normalized as “1” was used as a reference. Error bars indicate standard deviations of independent biological replicates (*n* = 3). Data are given as means ± SD. *p < 0.05, **p < 0.01 compared with unstressed samples using Student’s *t*-test.

The number and distribution of environmental stress-related and hormone-responsive elements in Snf2 genes are shown in Figures 5A,B, Supplementary Figures 3A,B, and Supplementary Table 5. The abundant elements were dehydration-responsive element, MeJA responsive element, stress-responsive element, MYB binding site for drought, and ABA-responsive elements. To explore the possible involvement of Snf2 genes in abiotic stress response, we analyzed the expression patterns of eight genes in the seedling after low temperature (4°C) and ABA treatments. The expressions of *OsCHR711*, *OsCHR715*, *OsCHR727*, *OsCHR728*, and *OsCHR732*, which have two or more LTR elements in each promoter region, were significantly increased within 3 h after cold stress treatment by qRT-PCR (Figure 5C). Similarly, the promoter regions of *OsCHR703*, *OsCHR715*, *OsCHR728*, *OsCHR733*, and *OsCHR735* have four or more ABRE elements, and the expression level of these genes was remarkably increased within 6 h after ABA treatment (Figure 5D). However, the promoter regions of *OsCHR707*, *OsCHR717*, *OsCHR724*, *OsCHR725*, and *OsCHR726* have one or no Auxin-responsive element, and these genes responded slowly or not to IAA treatment (Supplementary Figure 3C). These results suggested that Snf2 genes might be involved in plant response to abiotic and hormones stresses, and the *cis*-acting elements in the promoter regions were coordinated with the expression of genes.

### Most Snf2 Genes Respond Quickly After the *Magnaporthe oryzae* Infection

The above result shows that Snf2 genes have the most abundant stress response elements in the putative promoter regions. Then what specific roles do Snf2 genes play in response to stress resistance? The previous researches revealed that *OsCHR725* (*OsBRHIS1*) played a critical role in the SA-independent disease resistance by suppressing the innate immunity (Li et al., 2015), and *OsCHR706* (*OsALT1*) negatively regulated alkaline tolerance (Guo et al., 2014). These observations had prompted us to analyze the differential expression pattern of Snf2 genes under biotic and abiotic stress conditions. We analyzed the expression levels of 40 Snf2 genes in two-week-old seedlings at seven different time points for biotic (*M. oryzae*) infection and at four different time points for abiotic (salt and drought) stresses.

**FIGURE 6:**
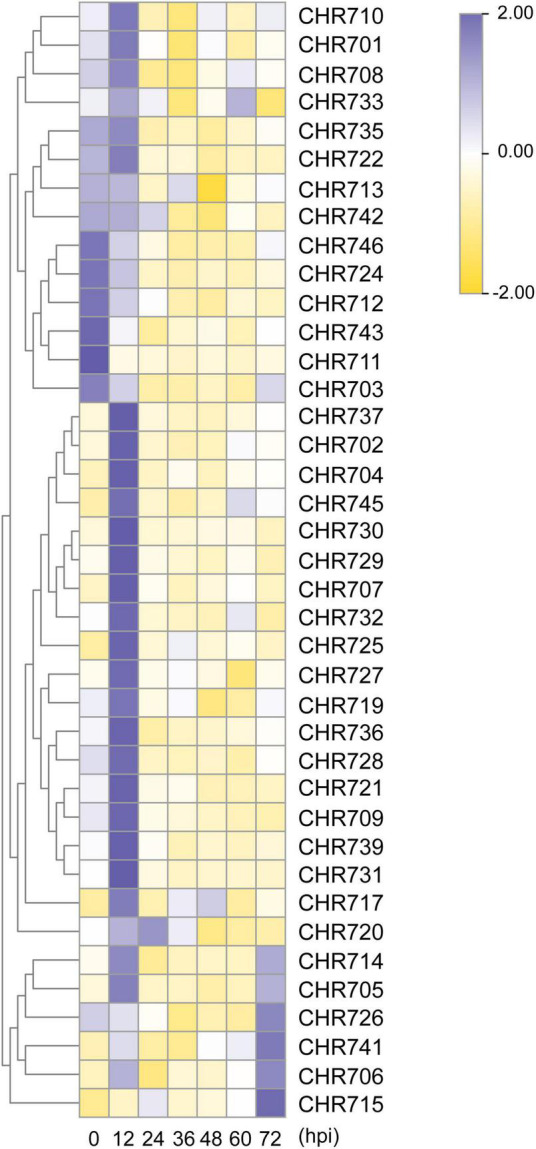
The relative expression level of Snf2 genes under *M. oryzae* stress. The expression analysis of Snf2 genes under the infection causal agent of rice blast. Heat map representing the temporal expression patterns was constructed by the heatmap packages in R. The data was normalized using *OsUBQ5* as the internal reference gene. hpi, hours post-inoculation.

When plants were exposed to environmental stresses, various physiological and biochemical responses were induced, leading to changes in gene expression (Ma et al., 2011). Briefly, after the plants were sprayed with *M. oryzae* GUY11 isolate, the spores of *M. oryzae* formed appressoria at 12 hpi and formed invasive hyphae at 24 hpi. Genes showing an up- and down-regulation were at least two-fold and significant difference compared with unstressed samples considered to further analysis compared to controls. Then, compared with the control plant, a total of 21 (52.5%) Snf2 genes were upregulated at 12 hpi, except for *OsCHR720*, which was upregulated at 24 hpi, and 12 (30%) genes were downregulated at all hpi (Figure 6 and Supplementary Figure 4). *OsCHR725* (*OsBRHIS1*) was upregulated fourfold at 12 hpi, which could be consistent with its function in regulating innate immunity, and *OsCHR707* was the most upregulated 12.6 folds than the control plants at 12 hpi, suggesting that most of Snf2 genes might play critical roles in regulating plant biotic stress and could respond quickly after the *M. oryzae* infection (Supplementary Table 6).

### Most Snf2 Genes Were Upregulated Under Salt and Drought Treatments

Snf2 family proteins in *Arabidopsis* have been reported to play an important role in salt and drought stresses (Mlynarova et al., 2007). As described above, there were a lot of *cis*-acting elements related to stress response (dehydration-, drought-, and stress-responsive element) in the predicted promoters of Snf2 family genes (Figure 5 and Supplementary Figure 3). Then what is the expression patterns of Snf2 gene in response to salt and drought in rice ?

**FIGURE 7:**
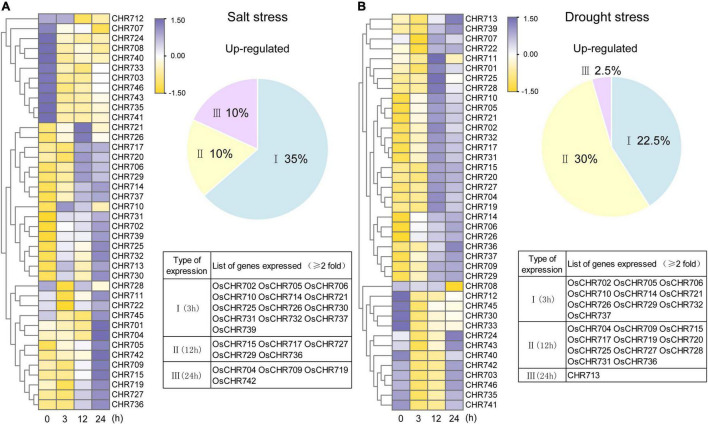
Expression analysis of Snf2 genes under salt and drought stresses. The temporal expression patterns of Snf2 genes under salt **(A)** and drought **(B)** stress treatments were presented by a heat map using the heatmap packages in R. The qRT-PCR values were normalized using *OsUBQ5* as the internal reference gene and that of the unstressed samples using the ΔΔC_T_ method. The percentage of genes upregulated (twofold and significant difference than the unstressed samples) under salt and drought treatments is represented in the pie chart beside the corresponding heat maps. The genes were annotated as I (3 h response), II (12 h response), and III (24 h response) types based on their time point of up-regulated expression. The names of the genes are provided in the forms.

The expression profiles of Snf2 genes were displayed in Figure 7 under salt (200 mM) and drought stresses by qRT-PCR analysis. Genes showing an up- and downregulation were at least two-fold and significant difference compared with unstressed samples considered to further analysis compared to controls. Based on expression level, we divided the up-regulated genes into I (response at 3 h), II (response at 12 h), and III (response at 24 h) types (Figure 7). As indicated in the pie chart, about 55% of the total genes were upregulated under salt and drought treatments, and 22–35% genes were upregulated expression responses at 3 h (Type I). Salt, however, induced 10% type II and type III responsive genes’ expression, whereas, drought induced type II genes (30%) and type III genes (2.5%). The list of the expressed genes has been provided in Figures 7A,B. Most of the type I genes continued their expression till 24 h after the treatment, except for *OsCHR702* and *OsCHR732*, which showed a split at subsequent time points (Supplementary Figures 5A,C). Both salt and drought stresses are associated with water management in plants, and then the results for these two stimuli were integrated. A total of 11 and 10 Snf2 genes were up-regulated during all experimental time points under salt and drought treatments, respectively. In addition, 9 and 4 genes were downregulated at all treatment time points, suggesting that they might play a negative role in salt and drought stresses (Supplementary Figures 5B,D). *OsCHR721* and *OsCHR714* were the most up-regulated, 22.5- and 39.5-folds than the control plants under salt and drought stresses, respectively (Supplementary Table 7). It was noteworthy that the seven genes (*OsCHR705*, *OsCHR706*, *OsCHR710*, *OsCHR714*, *OsCHR721*, *OsCHR726*, and *OsCHR737*) were upregulated and one gene (*OsCHR712*) was downregulated under both salt and drought stresses (Supplementary Figure 6). These results indicated that Snf2 genes might play a significant role in abiotic stress pathways and could be an excellent resource for evaluating stress tolerance in rice.

### Loss-Function of *OsCHR726* Decreases Resistance of Transgenic Rice to Salinity Stress

**FIGURE 8:**
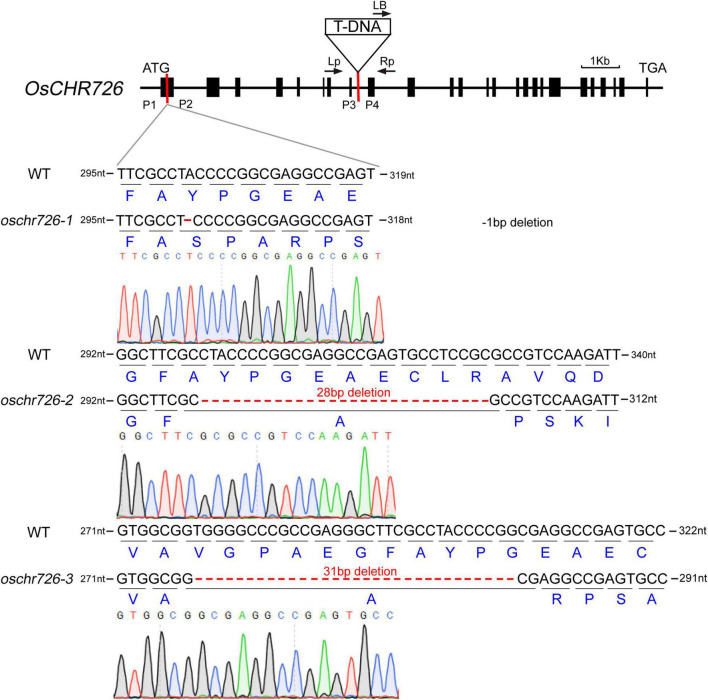
Genotyping analysis of *oschr726*. The triangle indicated the T-DNA insertion site in gene structure of *OsCHR726*. The arrows indicated the location of the primers for genotyping study of the T-DNA mutant. P3 and P4 were the positions of primers for identifying the transcription level of *OsCHR726* in the T-DNA mutant by qRT-PCR. The primers of P1 and P2 were used for identifying the CRISPR/Cas9-mediated mutations in *OsCHR726* in the T2 generation. The diagrams display the sequence results of the three CRISPR mutations. The black letters indicated the base pairs with WT in the target site. The dotted lines indicated nucleotide deletions, and dark blue letters indicated the amino acids encoded by the WT and *oschr726* sequences.

According to the above results, *OsCHR726* was upregulated under salt and drought stresses at all time points. Then what function does *OsCHR726* play in response to stress resistance? Here, we characterized a T-DNA insertion mutant, *oschr726-T* (2C-10296.L), in Dongjin background (Figure 8), which was identified in SIGnAL database (see footnote 1). The homozygous plants for the T-DNA insertion were identified by PCR, and the *OsCHR726* transcription level in *oschr726-T* was assessed by qRT-PCR (Supplementary Figure 7).

Apart from this, we subsequently performed *OsCHR726* CRISPR mutations using the CRISPR/Cas9 gene-editing system (Ma et al., 2015b). The primer P1 and P2 were used to identify whether mutations were introduced in the targeted sites by PCR and sequencing. Three homozygous mutant lines were obtained from T2 generation containing different mutations in the first exon of *OsCHR726* and named *oschr726-1*, *oschr726-2*, *oschr726-3*, respectively (Figure 8). *oschr726-1* generates a frameshift mutation resulting from a 1 bp (A) deletion at position C302 bp of the coding sequence (CDS). *oschr726-2* contains a 28-bp deletion at positions C300-327 bp, and *oschr726-3* contains a 31 bp deletion at positions C278-308 bp. The protein sequences of OsCHR726 were altered and its function was knocked out in the three mutant lines. The plant architecture of *oschr726* mature plants were similar with the wild-type. However, the seed setting of *oschr726* was reduced. However, it is unknown whether *OsCHR726* has functions in the regulation of male or female gametophyte.

**FIGURE 9:**
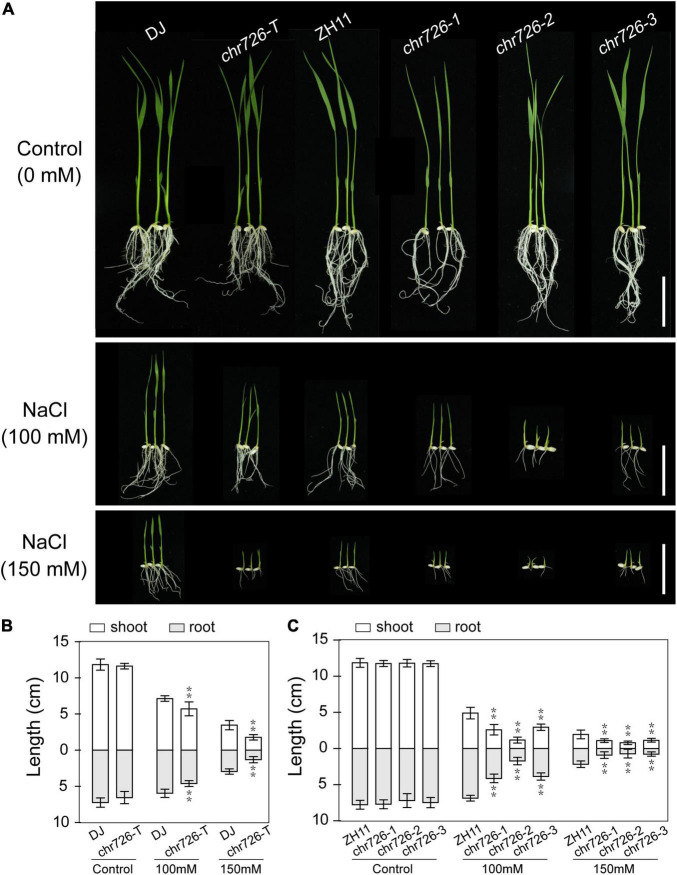
*oschr726* is sensitive to salt stress treatment. **(A)** The performance of *oschr726* seedlings compared with WT on media containing 0, 100, 150 mM NaCl. Bar = 5 cm. **(B)** The shoot and root lengths of 7-day-old seedlings were analyzed on WT (DJ) and T-DNA mutant. **(C)** The shoot and root lengths of 7-day-old seedlings were analyzed on WT (ZH11) and CRISPR/Cas9-mediated mutations. *n* = 20, Two asterisks (**p < 0.01, Student’s *t*-test) represent significant differences between the wild-type and mutants.

To confirm the effects of disrupted *OsCHR726* function on salt tolerance, the salt responses of *OsCHR726* mutation were examined on a solid medium. After germination for 2 days on half MS medium, seeds were transferred to a medium containing 0, 100, and 150 mM NaCl. The *oschr726-T* and *oschr726-1/2/3* seedlings did not differ from DJ and ZH11 when grown on the control medium, respectively (Figure 9A). However, the *OsCHR726* mutations showed greater growth inhibition with salt than wild-type seedlings depending on NaCl concentration (Figure 9A). The shoot and root length in the mutant lines were all significantly lower than the relevant wild-type (Figures 9B,C), indicating that *OsCHR726* might play a positive role in salt tolerance.

## Discussion

### Differential Expression Patterns of Snf2 Genes in Various Organs

In this study, we systematically analyzed the spatio-temporal expression patterns of Snf2 genes during reproductive development and stress treatments. Many genes displayed specific expression patterns at different developmental stages of various tissues. Most of the genes were highly expressed in YL, SAM, and OV (Figure 1). The loss-function of *BRM* reduced root length and plant height (Farrona et al., 2004), curly leaves (Hurtado et al., 2006), early flowering (Farrona et al., 2011) in *Arabidopsis*. *OsCHR707*, the homologous gene of *BRM*, was preferentially expressed in YL, P1, P2, and Ov5-6 (Figures 1A, 3C), suggesting that *OsCHR707* might participate in leaf or reproductive development. The deficient function of *AtCHR4* plants displayed faster leaf production in later rosette growth stages, more cauline leaves, and larger shoot apical meristems, and *AtCHR4* played a positive regulator of floral transition (Sang et al., 2020). Similarly, *OsCHR729*, a homologous gene of *AtCHR4*, was predominantly expressed in YL, SAM, An5-6 (Figures 1A, 2B), and mutation of *OsCHR729* exhibited rolled leaves, repressed stem elongation, and reduced chlorophyll contents (Hu et al., 2012). Apart from these, *OsCHR729* regulated seedling and root development via gibberellin and auxin-related signaling pathways, respectively (Ma et al., 2015a; Wang et al., 2016). *OsCHR721*, a nuclear protein belonging to the SMARCAL1 subfamily in Snf2, was predominantly expressed in SAM, P1, P2, An5-6, and Ov5-6 (Figures 1A, 2B, 3B). *OsCHR721* interacts with OsRPA1a (replication protein A), which is involved in DNA repair, and plays a crucial role in both male and female reproductive development (Zhang et al., 2020b). The above results suggest that the expression patterns of genes are coordinated and closely related with their function. *OsCHR722*, *OsCHR742*, *OsCHR735*, and *OsCHR746* were specifically expressed in SAM, and *OsCHR715*, *OsCHR703*, *OsCHR717* were specifically expressed in Sti, S2 and S3, respectively (Figures 1A,B). Interestingly, *OsCHR709*, *OsCHR711*, *OsCHR720*, *OsCHR730*, *OsCHR735*, *OsCHR739*, *OsCHR742*, *OsCHR743*, *OsCHR745*, and *OsCHR746* were specifically expressed in the early development of pistil (Ov1). In general, the relative expression profiles of Snf2 genes extent our understanding in vegetative and reproductive development.

### Expression Regulation of Snf2 Genes Under Biotic and Abiotic Stresses

Increasing evidence have demonstrated that some Snf2 genes are induced or inhibited expression by abiotic stress (Hu et al., 2013), and *OsALT1* (*Alkaline Tolerance 1*, *OsCHR706*) improves tolerance to alkaline stress (Guo et al., 2014). We all know that high salinity and drought are two major stress factors that can seriously affect plant growth, development, and crop yield. When plants were exposed to environmental stresses, various physiological and biochemical responses were induced, and gene expression programs rapidly adapted to adverse situations. To investigate the expression pattern of Snf2 genes under biotic and abiotic stresses, we predicted the *cis*-acting elements of hormone-responsive and environmental stress-related in the promoter regions. We performed qRT-PCR analysis of *M. oryzae* infection, salt, and drought stress at different experimental timelines.

The most elements in Snf2 putative promoter were dehydration-responsive element, MeJA-responsive element, stress-responsive element, MYB-binding site for drought element, and ABA-responsive element. Interestingly, there were 15 stress-responsive elements in the promoter of *OsCHR725* (Supplementary Figure 3), which was significantly upregulated at the 12 hpi of *M. oryzae* (Figure 6 and Supplementary Figure 4) and all treatment timelines of salt stress (Figure 7A and Supplementary Figure 5A). Previous studies have shown that *OsCHR725* (*OsBRHIS1*) plays a critical role in the SA-independent disease resistance (Li et al., 2015), suggesting that the *cis*-acting elements in the promoter are coordinated with the gene expression and function. *AtCHR19* acts as a transcriptional repressor and contributes to plant pathogen resistance (Kang et al., 2022). *OsCHR714*, the homologous gene of *AtCHR19*, was significantly upregulated at the 12 hpi of *M. oryzae* and all treatment timelines of salt and drought stress. This implied that this gene probably possesses similar characteristic functions on stress tolerance. A total of 20 genes were upregulated at 12 hpi but downregulated at subsequent time points under *M. oryzae* infection, indicating that these genes were possibly required for initial stress responses. *OsCHR726* was upregulated under salt stress during the treatment timelines, and *OsCHR726* loss-of-function mutants were hypersensitive to salt stress. Taken together, the expression profiles of Snf2 under biotic and abiotic stresses will provide a valuable resource for investigating stress tolerance in rice.

### Chromatin Remodeling Factors With Epigenetic Regulation

In this study, the subcellular localizations of the representative Snf2 proteins were mainly in the nucleus (Figure 4). Then Snf2 family proteins, as CHRs, regulate diverse aspects of DNA events such as replication, transcription, homologous recombination, and DNA repair (Hu et al., 2013). Chromatin structure plays a significant platform in regulating gene expression, whose mechanisms are associated with epigenetic regulation: DNA methylation, histone modifications and variants, and chromatin remodeling (Knizewski et al., 2008; Clapier and Cairns, 2009). The epigenetic mechanisms in plant stress response have been discussed in multiple reviews (Chinnusamy and Zhu, 2009; Asensi-Fabado et al., 2017; Chang et al., 2020; Song et al., 2021). However, the function of chromatin remodeling in plant stress responses has not been in-depth analyzed recently.

DRD1, a Rad 54-like family member, and DDM1, a member of Snf2-like protein, are involved in DNA methylation (Kanno et al., 2004). Functional deficiency of *DDM1* leads to promoting a decrease of methylation from some repeats and solid transcriptional activation of TEs (Lippman et al., 2004). *OsDDM1* genes (*OsDDM1a/OsCHR746* and *OsDDM1b/OsCHR741*) are essential for CHG and CG methylation in heterochromatic regions and CHH methylation in euchromatic regions (Tan et al., 2016). In addition, *OsCHR729* recognizes and modulates the H3K4me3 and H3K27me3 levels to control target genes involved in plant development (Hu et al., 2012). *OsCHR725* (*OsBRHIS1*), the ortholog to *AtCHR28*, can repress innate immunity for the reason of binding to specific histone H2A and H2B variants near the promoter regions of defense genes (Li et al., 2015). *OsCHR726* is hypersensitive to salt stress, but it is still unknown which epigenetic mechanism is involved. Current research on CHRs is mainly performed in the model plant *Arabidopsis* and a few crop species. We believe that more studies will be conducted to analyze the stress adaptation of CHRs in crops. Here, we provide systematical expression profiles of Snf2 family genes during the reproductive development and stress response. Our data suggest that Snf2 family genes might play a significant role in plant development and stress responses and be valuable for developing new plant varieties with enhanced stress tolerance.

## Accession Number

The cDNA and genomic DNA sequence data of *OsCHR726* can be found in the Rice Genome Annotation Project data libraries under accession number LOC_Os07g40730.

## Data Availability Statement

The datasets presented in this study can be found in online repositories. The names of the repository/repositories and accession number(s) can be found in the article/Supplementary Material.

## Author Contributions

YQ and HZ conceived the initial screening and research plans. HZ designed the experiments. MG, ZH, WZ, ZS, CS, and MY performed most of the experiments. WZ, CS, MY, and DT extracted the RNA. MM, MG, and WZ performed the qRT-PCR and analyzed the data. ZS performed the heatmap. ZH performed the CRISPR mutations. YQ, HZ, and MG wrote the manuscript with the contributions of all the authors. All authors contributed to the article and approved the submitted version.

## Conflict of Interest

The authors declare that the research was conducted in the absence of any commercial or financial relationships that could be construed as a potential conflict of interest.

## Publisher’s Note

All claims expressed in this article are solely those of the authors and do not necessarily represent those of their affiliated organizations, or those of the publisher, the editors and the reviewers. Any product that may be evaluated in this article, or claim that may be made by its manufacturer, is not guaranteed or endorsed by the publisher.

## Footnotes

1
http://signal.salk.edu/cgi-bin/RiceGE

2
http://gsds.gao-lab.org/index.php

## Supplementary Material

Supplementary Figure 1 The relative expression level of Snf2 gene family in the stamen. **(A)** Preferentially expressed Snf2 genes at the An1, An5, and An6 stages. **(B)** Preferentially expressed Snf2 genes at the An4∼An6 stages. **(C)** The irregularly expressed Snf2 genes in the different developmental stages of stamen. An1-5, the stamen of 2–3, 3–4, 4–5, 5–6, and 6–7 mm spikelet, respectively; An6, the stamen in the spikelet before flowering. The *y*-axis is the relative expression level of the gene compared to *OsUBQ5* in different developmental stages of stamen using the qRT-PCR. Error bars indicate standard deviations of independent biological replicates (*n* = 3). Different letters denote significant difference at *P* < 0.05 according to ANOVA in combination with Duncan’s multiple range test.

Supplementary Figure 2 The relative expression level of Snf2 gene family in the ovary. **(A)** The highly expressed Snf2 genes at the Ov1 and Ov4∼Ov6 stages. **(B)** The highly expressed Snf2 genes at the Ov4 stage. **(C)** The irregularly expressed Snf2 genes in all developmental stages of the ovary. Ov1-5, the ovary of 2–3, 3–4, 4–5, 5–6, and 6–7 mm spikelet, respectively; Ov6, the ovary of the spikelet before flowering. The *y*-axis is relative expression level of the gene compared to *OsUBQ5* in different developmental stages of ovary using the qRT-PCR. Error bars indicate standard deviations of independent biological replicates (*n* = 3). Different letters denote significant difference at *P* < 0.05 according to ANOVA in combination with Duncan’s multiple range test.

Supplementary Figure 3 Prediction of *cis*-acting elements of environmental stress-related and hormone-responsive in Snf2 genes. **(A)** Numbers of *cis*-acting elements detected in the promoter regions (sequence retrieved from about ≤ 2 kb upstream region). The *cis*-acting elements were divided into 11 types. **(B)** Quantity, kind and position of environmental stress-related and hormone-responsive elements in the putative promoters of genes in **(A)**. **(C)** The qRT-PCR analysis of Snf2 genes under IAA treatment. Error bars indicate standard deviations of independent biological replicates (*n* = 3). Data are given as means ± SD. **p* < 0.05, compared with unstressed samples using Student’s *t*-test.

Supplementary Figure 4 Statistical analysis of Snf2 genes under biotic stress. The percentage of genes up- and down-regulated (at least twofold and significant difference) in 12 hpi than the unstressed samples, and others under the infection of *Magnaporthe oryzae* (*M. oryzae*) is represented in the pie chart. The corresponding number and the list of genes is mentioned in the figure. hpi, hours post-inoculation

Supplementary Figure 5 Statistical analysis of Snf2 genes under abiotic stress. Venn diagrams using jvenn tools (http://jvenn.toulouse.inra.fr/app/usermanual.html) showing that the number of genes up-regulated **(A,C)** and down-regulated **(B,D)** were at least twofold and significant difference than the unstressed samples in different time points under salt and drought treatment. The corresponding number and the list of genes under each time point and in combination are mentioned in the figure.

Supplementary Figure 6 Up- and down-regulated genes under both salt and drought stress. **(A)** Up-regulation genes under both salt and drought stress. **(B)** Down-regulation genes under both salt and drought stress.

Supplementary Figure 7 Genotype identification of *oschr726* T-DNA mutant. **(A)** Genotyping of T-DNA insertion plants. **(B)** Identifying the transcription level of *OsCHR726* in the T-DNA insertion plants.
