# Supplementary figures and images for: Comparative Expression Profiling of Snf2 Family Genes During Reproductive Development and Stress Responses in Rice
Source: Front Plant Sci. 2022 May 31;13:910663. doi: 10.3389/fpls.2022.910663 (PMC9194907; doi:10.3389/fpls.2022.910663)

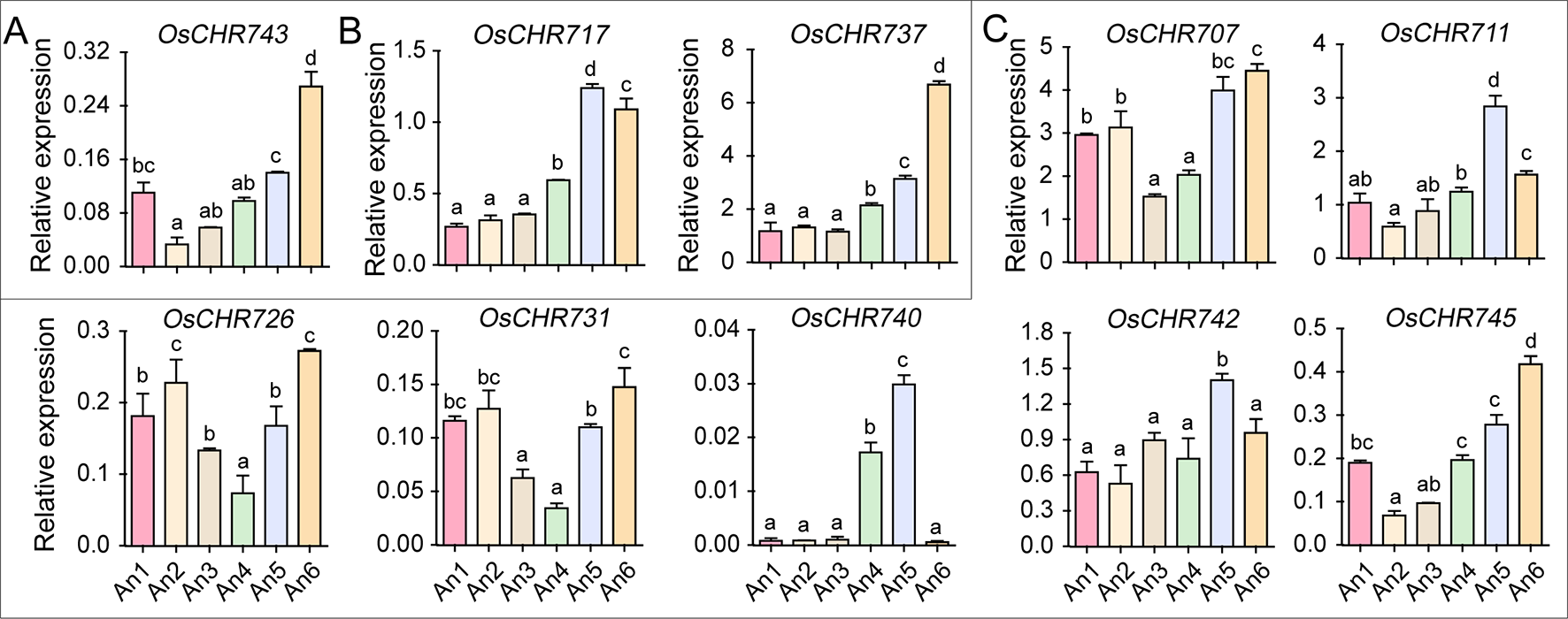

Supplement: Supplementary Figure 1 — The relative expression level of Snf2 gene family in the stamen. (A) Preferentially expressed Snf2 genes at the An1, An5, and An6 stages. (B) Preferentially expressed Snf2 genes at the An4∼An6 stages. (C) The irregularly expressed Snf2 genes in the different developmental stages of stamen. An1-5, the stamen of 2–3, 3–4, 4–5, 5–6, and 6–7 mm spikelet, respectively; An6, the stamen in the spikelet before flowering. The y-axis is the relative expression level of the gene compared to OsUBQ5 in different developmental stages of stamen using the qRT-PCR. Error bars indicate standard deviations of independent biological replicates (n = 3). Different letters denote significant difference at P < 0.05 according to ANOVA in combination with Duncan’s multiple range test. [file Image_1.TIF]

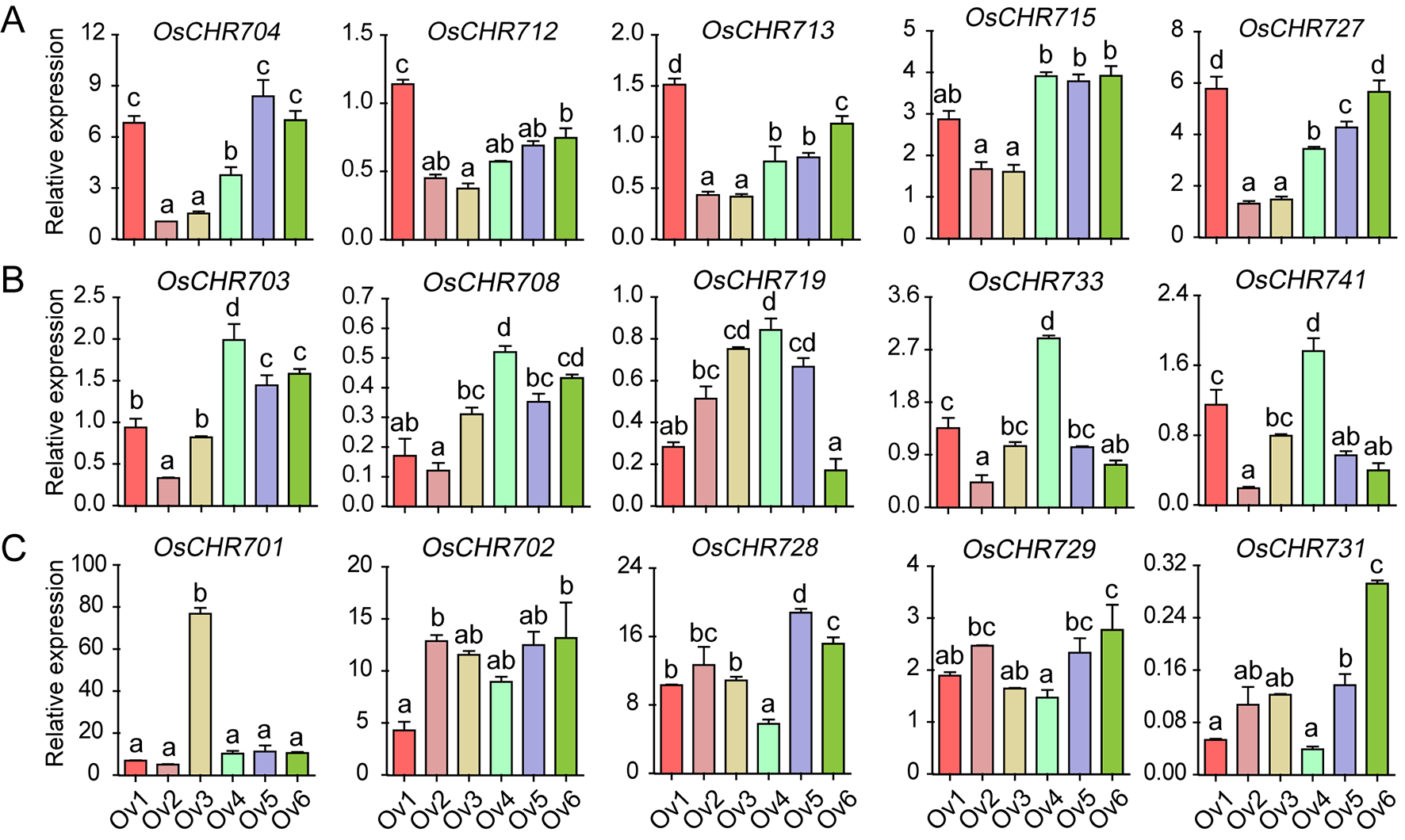

Supplement: Supplementary Figure 2 — The relative expression level of Snf2 gene family in the ovary. (A) The highly expressed Snf2 genes at the Ov1 and Ov4∼Ov6 stages. (B) The highly expressed Snf2 genes at the Ov4 stage. (C) The irregularly expressed Snf2 genes in all developmental stages of the ovary. Ov1-5, the ovary of 2–3, 3–4, 4–5, 5–6, and 6–7 mm spikelet, respectively; Ov6, the ovary of the spikelet before flowering. The y-axis is relative expression level of the gene compared to OsUBQ5 in different developmental stages of ovary using the qRT-PCR. Error bars indicate standard deviations of independent biological replicates (n = 3). Different letters denote significant difference at P < 0.05 according to ANOVA in combination with Duncan’s multiple range test. [file Image_2.TIF]

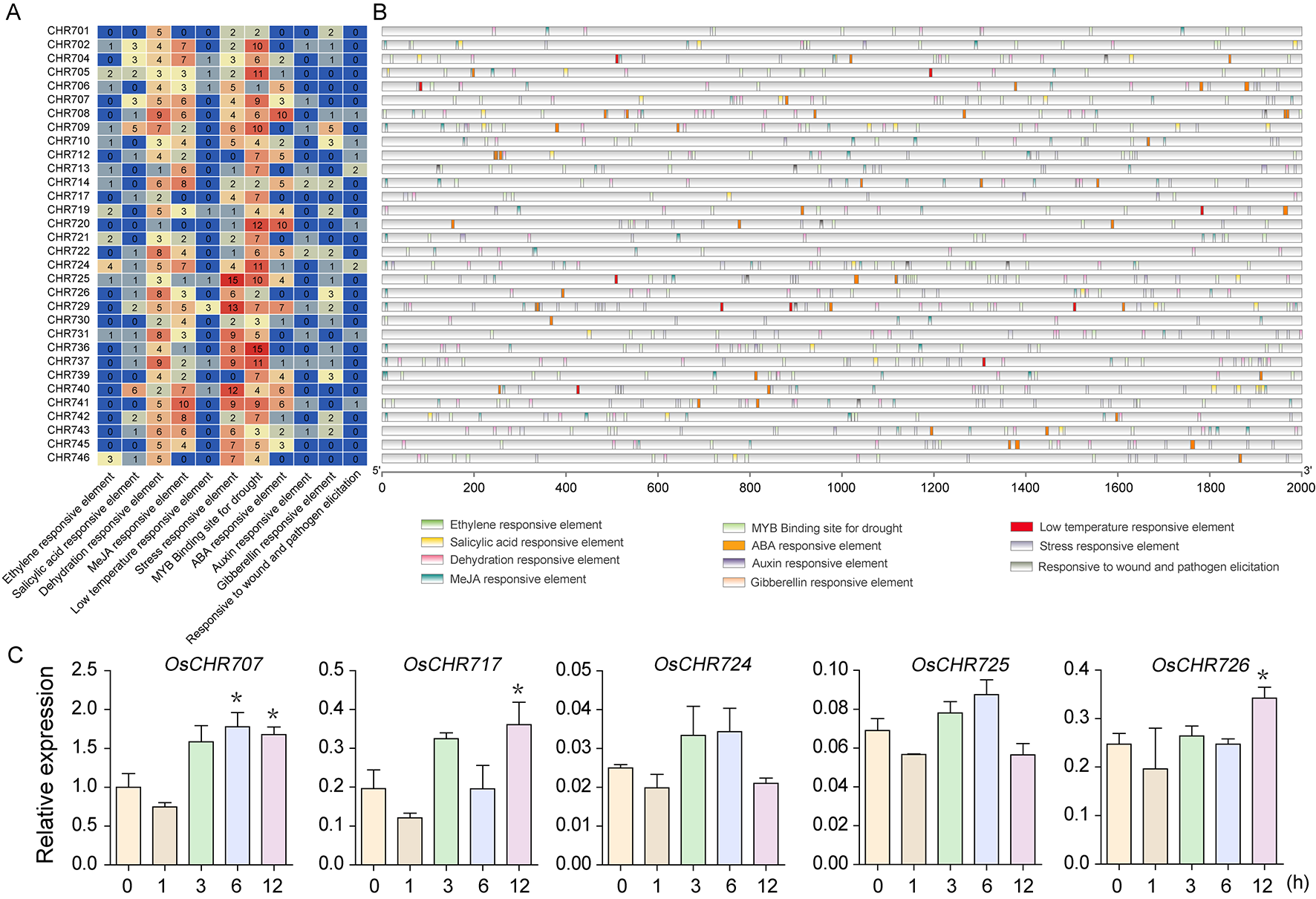

Supplement: Supplementary Figure 3 — Prediction of cis-acting elements of environmental stress-related and hormone-responsive in Snf2 genes. (A) Numbers of cis-acting elements detected in the promoter regions (sequence retrieved from about ≤ 2 kb upstream region). The cis-acting elements were divided into 11 types. (B) Quantity, kind and position of environmental stress-related and hormone-responsive elements in the putative promoters of genes in (A). (C) The qRT-PCR analysis of Snf2 genes under IAA treatment. Error bars indicate standard deviations of independent biological replicates (n = 3). Data are given as means ± SD. *p < 0.05, compared with unstressed samples using Student’s t-test. [file Image_3.TIF]

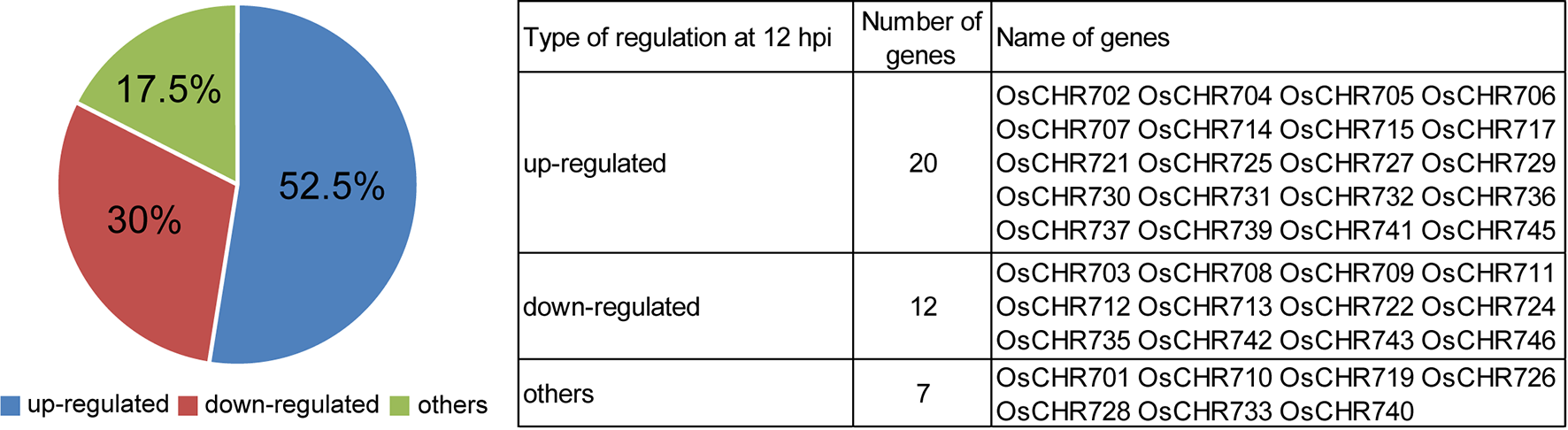

Supplement: Supplementary Figure 4 — Statistical analysis of Snf2 genes under biotic stress. The percentage of genes up- and down-regulated (at least twofold and significant difference) in 12 hpi than the unstressed samples, and others under the infection of Magnaporthe oryzae (M. oryzae) is represented in the pie chart. The corresponding number and the list of genes is mentioned in the figure. hpi, hours post-inoculation [file Image_4.TIF]

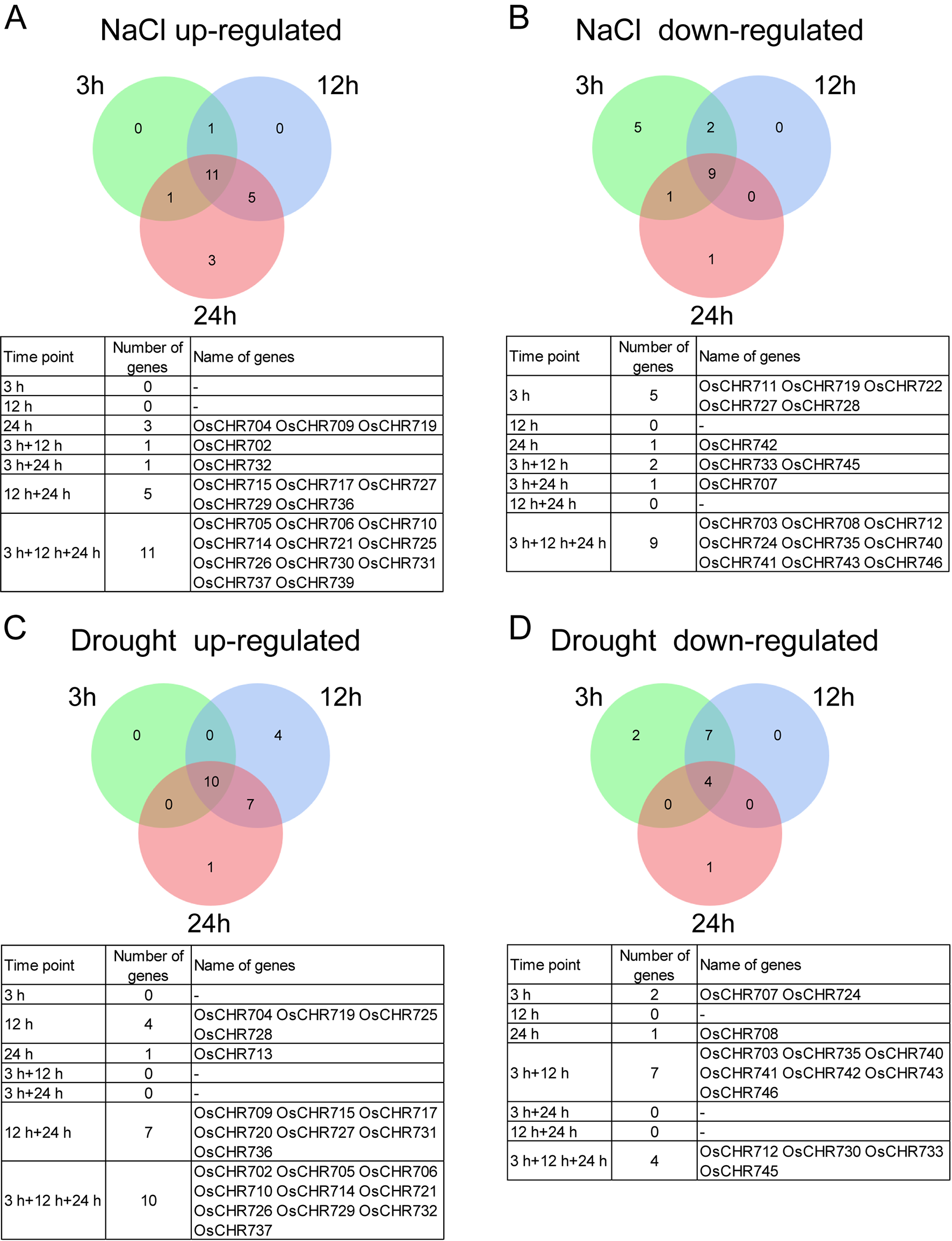

Supplement: Supplementary Figure 5 — Statistical analysis of Snf2 genes under abiotic stress. Venn diagrams using jvenn tools (http://jvenn.toulouse.inra.fr/app/usermanual.html) showing that the number of genes up-regulated (A,C) and down-regulated (B,D) were at least twofold and significant difference than the unstressed samples in different time points under salt and drought treatment. The corresponding number and the list of genes under each time point and in combination are mentioned in the figure. [file Image_5.TIF]

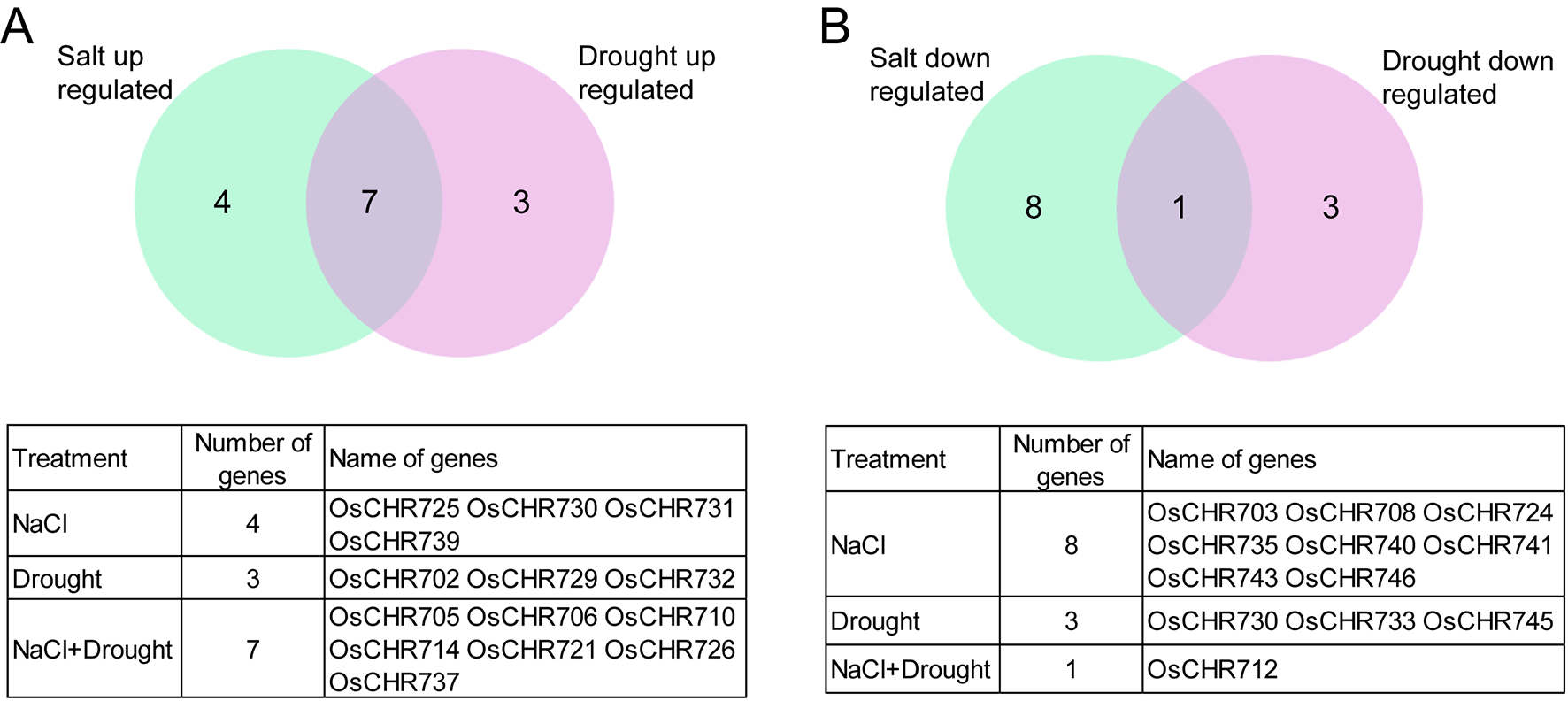

Supplement: Supplementary Figure 6 — Up- and down-regulated genes under both salt and drought stress. (A) Up-regulation genes under both salt and drought stress. (B) Down-regulation genes under both salt and drought stress. [file Image_6.TIF]

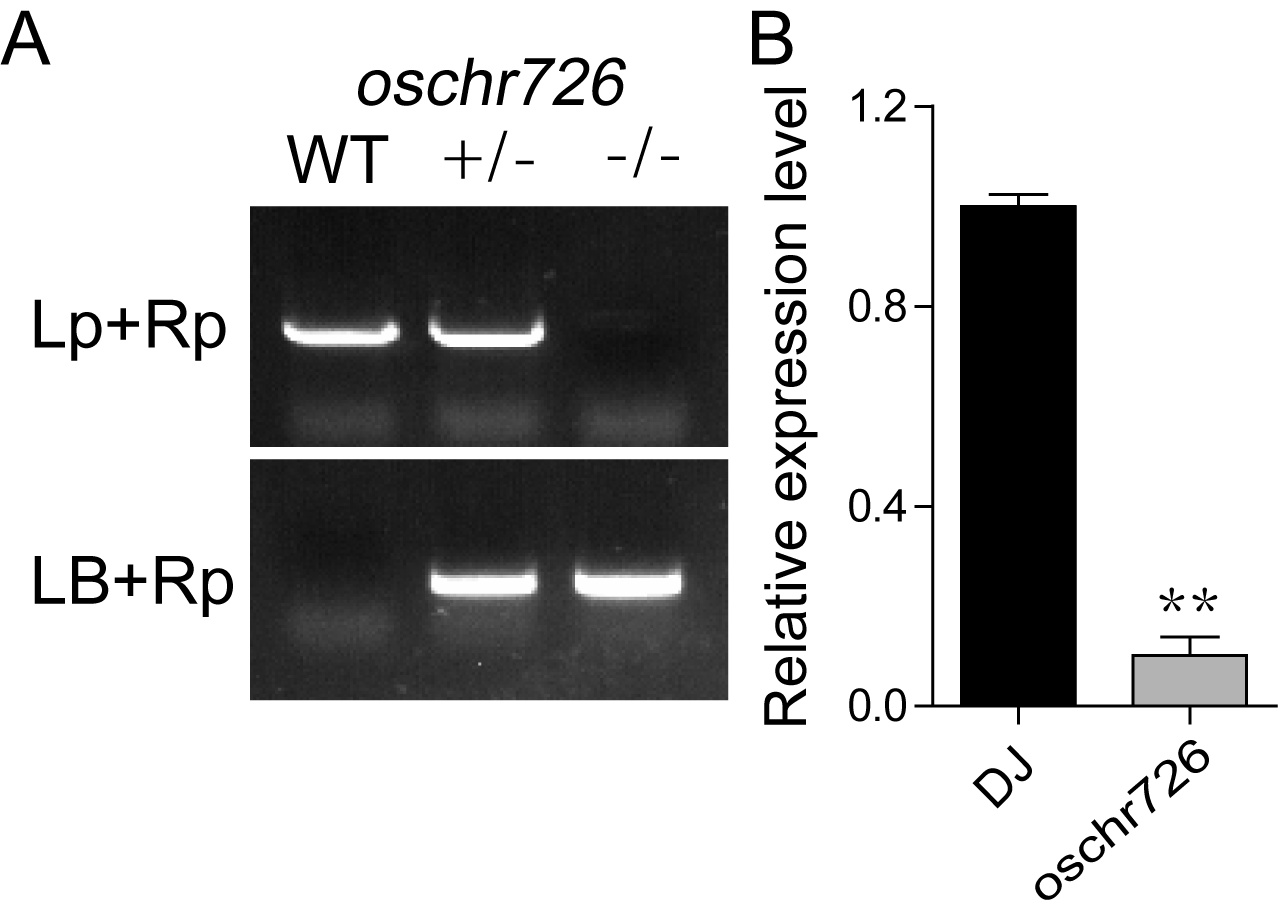

Supplement: Supplementary Figure 7 — Genotype identification of oschr726 T-DNA mutant. (A) Genotyping of T-DNA insertion plants. (B) Identifying the transcription level of OsCHR726 in the T-DNA insertion plants. [file Image_7.TIF]
